# TrWRKY41: A WRKY Transcription Factor from White Clover Improves Cold Tolerance in Transgenic Arabidopsis

**DOI:** 10.3390/plants14223493

**Published:** 2025-11-16

**Authors:** Meiyan Guo, Shuaixian Li, Jun Tian, Manman Li, Xiaoyue Zhu, Changhong Guo, Yongjun Shu

**Affiliations:** Key Laboratory of Molecular Cytogenetics and Genetic Breeding of Heilongjiang Province, College of Life Science and Technology, Harbin Normal University, Harbin 150025, China; 15145577725@163.com (M.G.); hsdlsx1201@stu.hrbnu.edu.cn (S.L.); tj2131228518@163.com (J.T.); limanman1208@stu.hrbnu.edu.cn (M.L.); zhuxiaoyue2001@126.com (X.Z.); kaku3008@hrbnu.edu.cn (C.G.)

**Keywords:** white clover, WRKY, overexpression, cold tolerance, *Arabidopsis thaliana*

## Abstract

*Trifolium repens* L. (white clover) is a widely distributed perennial legume, which is regarded as one of the most important forages for its high protein content and excellent palatability. Low temperature limits the distribution and productivity of white clover, thereby reducing its economic returns. WRKY transcription factors are key regulators in stress defense and are involved in multiple abiotic stress responses in plants. In this study, a cold inducible gene named *TrWRKY41* was cloned from white clover. The TrWRKY41 protein is predominantly localized in the nucleus and functions as a hydrophilic, acidic protein. Under cold stress, the overexpression plants had significantly higher chlorophyll (CHL) and proline (Pro) contents, significantly increased activities of catalase (CAT), peroxidase (POD), and superoxide dismutase (SOD), and malondialdehyde (MDA) content significantly decreased. Compared to wild-type *Arabidopsis thaliana*, *TrWRKY41*-overexpressing plants exhibited better cold tolerance. In addition, target genes downstream of the *TrWRKY41* transcription factor were predicted utilizing BLAST alignment and AlphaFold2 (version 0.2.0) software, the expression of six genes, including *AtCOR47*, *AtCOR6.6*, and *AtABI5*, was significantly up-regulated under cold stress. It suggests that *TrWRKY41* may enhance cold tolerance in Arabidopsis by activating the ICE-CBF-COR cascade. This study provides candidate genes for research on enhancing the cold tolerance of white clover.

## 1. Introduction

White clover (*Trifolium repens* L.) is a perennial legume widely utilized as forage and silage due to its high protein content, nutritional value, and palatability, supporting livestock industry development [1]. Due to its short growth cycle and strong regenerative capacity, and incorporating white clover into lawns enhances visual appeal, thus providing distinct landscaping value [2]. White clover is widely distributed in temperate and cool-temperate regions, where its growth is frequently compromised by abiotic stresses particularly cold stress [3]. These limitations severely constrain yield and reduce economic viability.

Cold stress is a key climatic factor affecting crop growth, development and geographical distribution [4]. Low temperature constitutes a major stress factor limiting crop productivity and development. Prolonged exposure to low temperatures triggers physiological and biochemical changes in plant cells. For example, damage to the plasma membrane; changes in membrane lipid composition and alterations in the photosynthetic apparatus and electron flow in plants [5]. In order to survive under cold stress conditions, plants have developed a system of mechanisms to protect themselves from negative environmental attacks [6].

In recent years, an increasing number of studies have focused on the cold tolerance of perennial leguminous plants, such as alfalfa (*Medicago sativa* L.) and *Glycyrrhiza glabra*. For instance, the expression of the *MsbHLH* genes was significantly up-regulated under cold stress, and it is involved in the cold stress signaling transduction network in alfalfa [7]. Similarly, NAC transcription factors widely regulate plant growth and development [8]. Fifteen *MsNAC* genes were identified to actively respond to cold, salt, and drought stress [9]. Under cold stress, the expression of *GgWRKY15*, *53*, and *54* was up-regulated, indicating a positive response to cold, whereas *GgWRKY14* and *40* were down-regulated, suggesting their negative regulatory roles [10]. These findings highlight the significance of TFs in mediating cold adaptation, and provide a foundation for studying TFs function in white clover.

Transcription factors (TFs) play an important role in this complex gene regulatory network [11]. Transcription factors broadly participate in cellular differentiation and developmental processes. By binding to cis-acting elements, TF genes regulate the expression of downstream target genes through signal transduction pathways, enabling adaptation to environmental stresses. Prominent TF families include bZIP, MYB, WRKY, and AP2/ERF [12,13,14,15]. Among the cold response pathways, the *Inducer of CBF Expression* (*ICE*), *C-repeat Binding Factors* (*CBF*), and *Cold-Regulated Genes* (*CORs*) are key genes in the cold stress response [16]. Together, these genes constitute the ICE-CBF-COR signal transduction pathway, which mediates plant cold tolerance by transmitting stress signals and reducing physiological damage [17,18]. *ICE* positively regulates and induces the expression of *CBF* (*DREB1* genes) [19]. Subsequently, CBF transcription factors bind to the cis-acting elements in the promoter regions of *COR genes* and induce their expression, thereby directly or indirectly regulating plant cold tolerance [20,21].

The WRKY family is found primarily in plants and constitutes one of the largest TF families in plants. WRKY transcription factors recognize and bind to the W-box cis-element (TTGACC/T) within target gene promoters, this molecular regulatory mechanism is capable of inducing changes at the plant physiological level, which enhances plant tolerance. WRKY proteins are zinc finger-type transcriptional regulators. Their name derives from the highly conserved WRKYGQK amino acid sequence at the N-terminus, and containing a conserved zinc finger motif (CX_4–5_CX_22–23_HXH or CX_7_CX_23_HXC) at the C-terminus [22]. Previous studies have shown that *CsWRKY2* plays an important role in the stress response to cold and drought stresses by participating in the downstream ABA signaling pathway [23]. *OsWRKY63* negatively regulates cold tolerance through the *OsWRKY63*-*OsWRKY76*-*OsDREB1B* cascade reaction in rice [24]. Banana fruit WRKY TFs improve crop cold tolerance by directly activating *NECD* expression [25]. Recent studies have demonstrated that overexpression of the grape WRKY transcription factor *VhWRKY44* increased cold tolerance in Arabidopsis plants [26]. These studies collectively demonstrate the critical functions of WRKY TFs in regulating plant growth and stress adaptation.

In recent years, with the continuous advancement of genome sequencing technology, WRKY TFs have been identified in an increasing number of species. In 2023, the WRKY transcription factor family was first identified in white clover [27]. The TrWRKY gene family comprises a total of 145 members. Based on the evolutionary relationship of At*WRKYs*, TrWRKY proteins can be divided into three major categories. Following 30 min of cold stress treatment, the expression of most *TrWRKY* genes was significantly upregulated, indicating a rapid response to cold stress. In addition, qRT-PCR analysis revealed that the expression levels of *TrWRKY41*, *TrWRKY79*, and *TrWRKY100* initially increased and then decreased after cold treatment, indicating their responsiveness to early cold stress.

Despite the identification of the TrWRKY gene family, the specific functions of individual members, such as TrWRKY41, in cold tolerance remain largely unexplored. Based on previous research results, we selected the *TrWRKY41* gene as the subject of our study to further explore its potential functions. To this end, we isolated *TrWRKY41* from white clover and then expressed it heterotopically in the model plant *Arabidopsis thaliana*. This study obtained three transgenic lines and preliminarily explored the mechanism of action of the *TrWRKY41* gene under cold stress. Our results provide potential genetic mechanisms for further studies of cold tolerance in white clover, as well as offering insights for molecular breeding.

## 2. Results

### 2.1. Cloning and Physicochemical Characterization of the TrWRKY41 Gene

**Figure A1:**
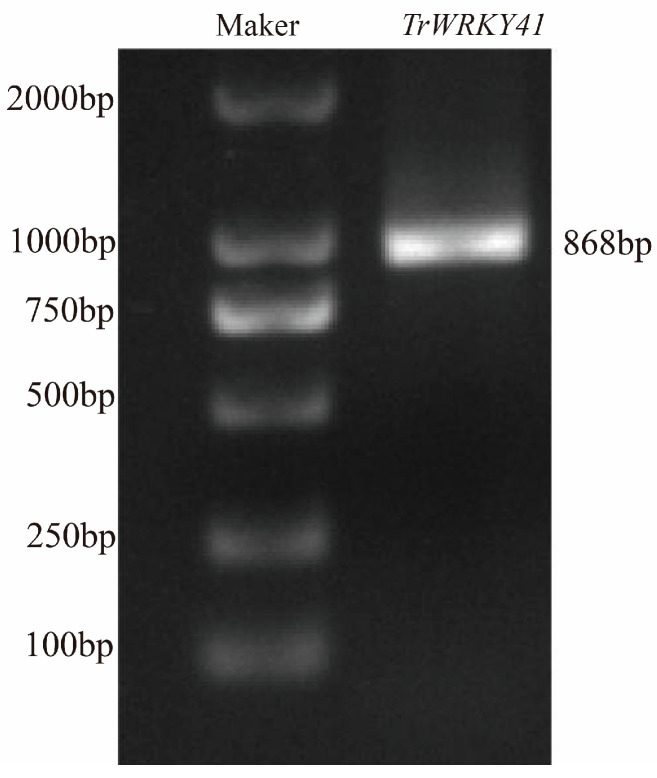
PCR amplification of the full-length *TrWRKY79* from white clover cDNA. Using white clover cDNA as the template, and *TrWRKY41* was amplified with gene-specific primers *TrWRKY41*-F and *TrWRKY41*-R. The PCR products were separated and verified by agarose gel electrophoresis.

*TrWRKY41* was cloned from the Haifa variety white clover. It was amplified from white clover cDNA with the specific primers *TrWRKY41*-F and *TrWRKY41*-R (Table A1). The PCR amplified *TrWRKY41* gene product was recovered and the product was detected by agarose gel electrophoresis. A specific target band of about 868 bp in length was obtained, and the results were as expected (Figure A1).

The physicochemical properties were further analyzed using the NCBI database as follows, the molecular formula of the protein encoded by *TrWRKY41* is C1405H2250N408O453S14; the molecular weight is 32,554.62 kDa, and the theoretical isoelectric point is 6.90; its instability index is 44.24, inferring that the TrWRKY41 protein is an unstable acidic protein. TrWRKY41 is composed of 20 amino acids, with serine (Ser), threonine (Thr), and leucine (Leu) being the three most abundant residues. The protein contains 33 positively charged residues (Arg + Lys) and 34 negatively charged residues (Asp + Glu). Additionally, it exhibits an aliphatic index of 72.47, a theoretical half-life of 100 h, and an average hydrophilicity (GRAVY) value of −0.664. Subcellular localization prediction was performed by WoLF PSORT. The results showed that TrWRKY41 protein accounted for 61.0%, 34.1% and 4.9% in the nucleus, cell membrane and cytoskeleton, respectively. ProtScale analysis of the *TrWRKY41*-encoded protein revealed a hydrophobicity range of −2.811 to 1.878. Combined with the results in ProtParam, the TrWRKY41 protein was predicted to be hydrophilic.

### 2.2. Overexpression of TrWRKY41 in Arabidopsis Increases Cold Tolerance

**Figure A2:**
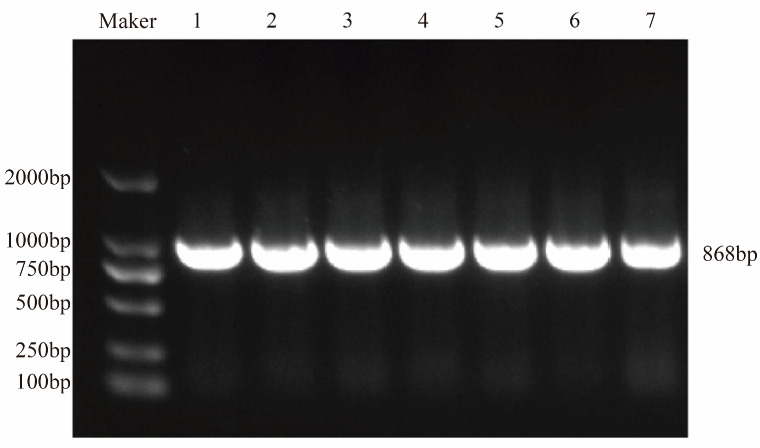
The purified PCR product was ligated into the pMD18-T Simple vector and transformed into *E. coli* DH5α competent cells. Single colonies were screened by PCR, and results showed amplicons of the expected size, consistent with sequencing data.

In order to reveal the potential biological functions of *TrWRKY41*, both pMD18T-*TrWRKY41* and pCAMBIA1300 plasmids were double-digested with PstI/BamHI restriction enzymes. Digestion products were gel purified separately. Subsequently, the digested fragments were ligated using T4 DNA ligase and transformed into *E. coli* DH5α competent cells via heat shock method. Plasmids extracted from positive clones were verified by agarose gel electrophoresis. The observed band sizes were as expected and consistent with the sequencing results (Figure A2). The correctly assembled plasmid was named pCAMBIA1300-*TrWRKY41*.

**Figure 1:**
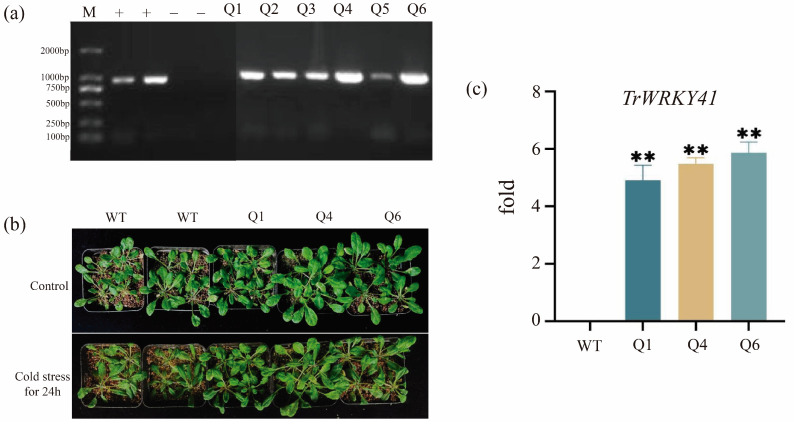
Phenotypic analysis and validation of T3 generation positive transgenic arabidopsis thaliana under cold stress. (**a**) Six independent T0 transgenic Arabidopsis lines (Q1–Q6) were identified by PCR using the 2000 bp Marker with negative (−) and positive (+) controls. Target bands were amplified in all transgenic lines but not in wild-type. (**b**) Phenotypes of wild-type and *TrWRKY41*-overexpressing homozygous lines (Q1, Q4, Q6) after 24 h cold stress treatment. (**c**) Expression of *TrWRKY41* in wild-type and transgenic lines (Q1, Q4, Q6) under cold stress, asterisks indicates a significant difference between groups (** *p* ≤ 0.01), and T-tests were used for intergroup comparisons. Expression data were calculated using the 2^−ΔΔCT^ method.

The recombinant plasmid was introduced into Agrobacterium tumefaciens GV3101 and then transformed into Arabidopsis. This resulted in six independent T0 transgenic lines (Figure 1). Genomic DNA extracted from leaves was subjected to PCR analysis. All six transgenic lines exhibited target bands matching the expected size, while no amplification was observed in wild-type plants (Figure 1). Three transgenic seedlings (Q1, Q4, and Q6) were finally obtained from *TrWRKY41*-overexpressing lines after multiple rounds of antibiotic screening (Figure 1). To further identify T3 generation positive seedlings, total leaf RNA was extracted and reverse transcribed to cDNA for qRT-PCR analysis. The results showed that the *TrWRKY41* gene was not expressed in the wild-type, whereas the expression of *TrWRKY41* in the transgenic lines (Q1, Q4, and Q6) was significantly higher than that in the control group, indicating that the gene was successfully transferred into Arabidopsis (Figure 1). The agarose gel electrophoresis results were further verified.

To verify the function of *TrWRKY41*, four-week-old Arabidopsis seedlings were subjected to cold stress (4 °C) for 24 h. Compared to wild-type controls, T3 generation transgenic lines (Q1, Q4, and Q6) exhibited significantly reduced leaf wilting and sustained growth vigor, whereas WT plants showed severe growth retardation and conspicuous leaf dehydration. These findings suggest that heterologous expression of the *TrWRKY41* enhances tolerance to cold stress in Arabidopsis.

### 2.3. Heterologous Expression of TrWRKY41 Gene Enhances Cold Tolerance in Arabidopsis

**Figure 2:**
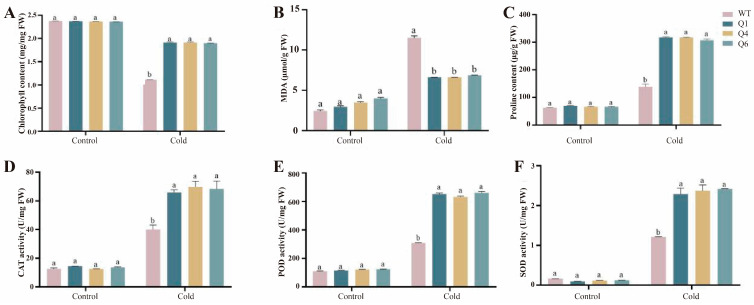
Physiological and biochemical indicators of transgenic Arabidopsis under cold stress. From left to right: wild-type Arabidopsis, homozygous lines Q1, Q4, and Q6. (**A**) Chlorophyll (CHL) content; (**B**) Malondialdehyde (MDA) content; (**C**) Proline (Pro) content; (**D**) Catalase (CAT) activity; (**E**) Peroxidase (POD) activity; (**F**) Superoxide dismutase (SOD) activity. Each sample was repeated three times. Duncan’s test was used for intergroup comparisons, with different letters indicating significant differences between different groups (*p* < 0.05).

Both transgenic homozygous lines and wild-type plants were subjected to cold stress (4 °C). Subsequently, chlorophyll (CHL), malondialdehyde (MDA), and proline (Pro) contents, as well as the enzymatic activities of catalase (CAT), peroxidase (POD), and superoxide dismutase (SOD), were measured (Figure 2). The results showed that the three overexpression lines Q1, Q4, and Q6, exhibited significantly enhanced cold tolerance compared to the wild-type. Specifically, the chlorophyll content was significantly higher than that of the wild-type under cold stress. This indicates that chlorophyll degradation is reduced in the transgenic lines, thereby enhancing plant cold tolerance. The three transgenic lines showed significantly reduced malondialdehyde contents than the wild-type (*p* < 0.05), indicating less membrane damage and superior cold stress tolerance. Moreover, the proline contents of Q1, Q4, and Q6 were significantly higher than wild-type (*p* < 0.05), suggesting that *TrWRKY41* was able to enhance cold stress tolerance by accumulating more proline. CAT, POD and SOD are crucial antioxidant enzymes in plants, which are capable of removing ROS generated under cold stress, and thus protecting plant cells from oxidative damage. Under cold stress, the activities of all three antioxidant enzymes were significantly increased in the transgenic plants. This suggests that the *TrWRKY41* gene removes ROS and reduces oxidative stress by increasing the activity of antioxidant enzymes. In summary, *TrWRKY41* enhances cold stress tolerance through changes in physiological and biochemical indicators in Arabidopsis.

### 2.4. TrWRKY41 Transcription Factor Binding Site and Structure Prediction

**Figure 3:**
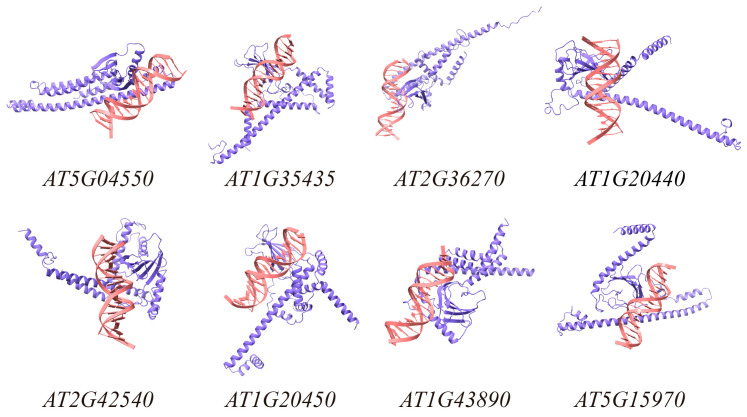
Structural prediction of TrWRKY41 protein and target genes. Purple represents TrWRKY41 protein, and pink represents the double strand of target gene DNA. The results were visualized with Chimera X (version 1.9) software.

To investigate the cold tolerance function of *TrWRKY41*, identifying its downstream target genes is necessary. In this study, we defined the 2000 bp upstream region of the Arabidopsis genomics as the promoter, and identified multiple potential WRKY binding sites by the core motif TTGAC [C/T]. The 20 sites with the highest frequency of occurrence were selected for the next study. (Table A2). Further analysis of the sequence shows that the WRKY core motif TTGAC [C/T] is highly enriched. This indicates that this motif is evolutionarily conserved, which is consistent with the results of previous studies. Subsequently, we performed BLAST alignments of Arabidopsis promoter sequences using the 20 motifs as query sequences. Finally, we randomly selected 30 sequences and used AlphaFold2 software to predict the structure of *TrWRKY41* target genes (Table 1). All TrWRKY41 protein-target gene structures had ipTM ≥ 0.8, indicating that the prediction results were highly reliable and credible [28]. Eight of the predicted results were selected for visualization using Chimera X (Figure 3). In summary, we found that *TrWRKY41* directly binds to the W-box region of the target gene promoter, forming a stable complex to mediate its regulatory effects in specific biological processes.

### 2.5. qRT-PCR Analysis of TrWRKY41 Downstream Target Genes Under Cold Stress

**Figure 4:**
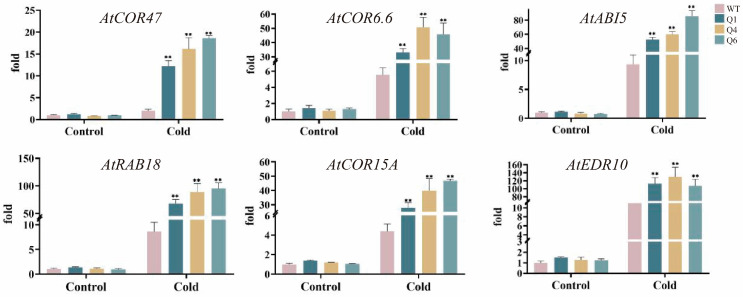
Expression analysis of downstream target genes in TrWRKY41-overexpressing lines under cold stress. *X*-axis: Control group and cold stress treatment group (with 3 replicates each). The different colors in the bar graph from left to right represent WT, Q1, Q4, and Q6 plants, respectively. *Y*-axis: Relative gene expression levels. The asterisk indicates a significant difference between the two groups. Statistical significance was determined using *t*-tests (** *p* < 0.01). WT expression was normalized. Expression levels were calculated using the 2^−ΔΔCT^ method.

WRKY transcription factors precisely activate or suppress downstream gene expression within complex regulatory networks. *AtCOR47*, *AtCOR6.6*, *AtABI5*, *AtRAB18*, *AtCOR15A* and *AtERD10* are reported cold stress response genes, and based on the prediction of cis-acting elements in the promoter region of Arabidopsis, we focused on studying these six key WRKY-regulated downstream genes. First, overexpressing *TrWRKY41* plants and the wild-type were subjected to cold stress (4 °C) for 24 h. The expression levels of these six key genes were significantly up-regulated in the *TrWRKY41* transgenic lines, and were significantly higher than in wild-type Arabidopsis (Figure 4). In summary, WRKY transcription factors respond to cold stress by specifically binding to W-box sequences in target gene promoters, thereby delicately regulating downstream genes expression. In contrast, overexpression of the *TrWRKY41* gene was able to significantly up-regulate the expression levels of cold stress-related genes, providing plants with a stronger low-temperature protection mechanism.

## 3. Discussion

During growth and development, plants are subjected to a variety of stresses. In order to survive and reproduce, plants have adapted to adversity through the evolution of their morphology and the regulation of their metabolic levels [29]. WRKY TFs are a key category of regulatory factors, which participate extensively in various physiological and biochemical processes. Previously, numerous studies have demonstrated the broad involvement of WRKY transcription factors in plant secondary metabolism. This involvement occurs in response to abiotic and biotic stresses, senescence, seed dormancy, and germination [30]. Therefore, studying the pivotal role of *TrWRKY* genes under cold stress is crucial for white clover breeding and crop improvement.

In this study, the *TrWRKY41* gene was successfully isolated from the white clover genome by homologous cloning. Its function was analyzed by Agrobacterium-mediated transformation into Arabidopsis plants. After 24 h of low-temperature treatment, the transgenic plants showed insignificant leaf damage and significantly better growth than the controls, and the expression of *TrWRKY41* gene was significantly increased, indicating the ability to respond positively to cold stress and less plant stress damage (Figure 1).

Cold stress also causes an influence on the metabolic level of the plant, so the measurement of physiological indicators is an important tool in the study of plant tolerance. Chlorophyll content is used to characterize plant growth [31]. Chlorophyll is the most essential tool for plants to capture light. Photosynthesis is highly susceptible to cold stress [32]. Studies have shown that cold stress inhibits PSII activity and reduces maximum photochemical efficiency in tobacco [33]. Excess malondialdehyde damages cell membranes. The more MDA accumulates, the more severe the membrane damage becomes [34]. Accumulation of osmotic substances contributes to plant tolerance to stress, and proline plays a critical role in regulating osmotic homeostasis [35]. Research has shown that free radicals and reactive oxygen species cause oxidative damage and tissue dysfunction [36]. Therefore, the removal of excess reactive oxygen species is essential to maintain the vital activities of the organism. CAT, POD and SOD are believed to scavenge reactive oxygen species and are considered as key physiological indicators in abiotic stress studies. Previously, the expression of the *CsWRKY21* gene in tea tree was significantly increased 6-times under cold stress [37]. Under cold stress conditions, the *PmWRKY57* gene overexpressing plants showed significantly higher superoxide dismutase and peroxidase enzyme activities, as well as higher proline content, compared with the wild-type. This significantly improved the cold tolerance in Arabidopsis [38]. Expression of *VvWRKY28* significantly increased under cold stress and enhanced cold tolerance in Arabidopsis plants. The activities of SOD, POD and CAT were increased while MDA content was decreased after treatment [39]. In this study, we determined the physiological indexes under cold stress. A number of studies have shown that *WRKY* genes can increase cold tolerance by increasing chlorophyll content, such as *VhWRKY44*, *BcWRKY46* and so on [26,40]. The results showed that the chlorophyll content of the transgenic lines was higher than the control, indicating that *TrWRKY41* may enhance the cold tolerance by regulating the expression of the *CHLH* genes in Arabidopsis (Figure 2) [41]. We also found that MDA content was reduced and proline content was raised in the transgenic plants, suggesting that the *TrWRKY41* gene enhances cold tolerance by enhancing cell membrane stability and regulating osmotic pressure. Moreover, CAT, POD and SOD activities were significantly higher than those of the control group, which was consistent with the results of previous studies (Figure 2). The above results emphasized that *TrWRKY41* gene plays an important role in plant response to cold stress.

Different WRKY transcription factors regulate different mechanisms. Certain WRKY transcription factors can bind to a cis-acting element (W-box) in the promoter region of a downstream target gene, thereby activating gene expression [42]. To clarify the regulatory mechanism of *TrWRKY41* and identify its downstream target genes, we extracted genome-wide promoters from Arabidopsis. We screened 30 downstream target genes of *TrWRKY41* by recognition and alignment of W-box elements in the promoter. Protein-DNA complex structures were predicted using AlphaFold2. Prediction accuracy was evaluated using the interface prediction template modelling (ipTM) score; all models achieved an ipTM ≥ 0.8, demonstrating their reliability [28]. Interestingly, the presence of W-box motifs in target gene sequences promoted interaction with *TrWRKY41* (Figure 3).

**Figure 5:**
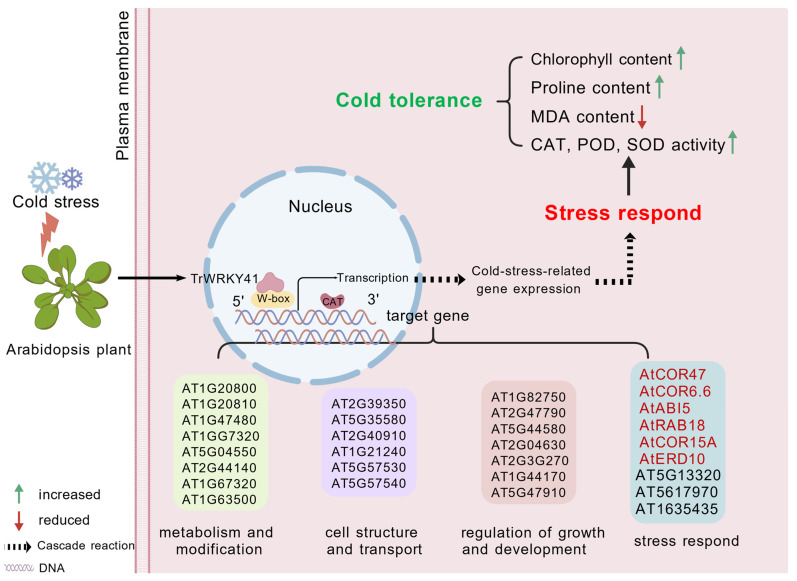
Mechanism of WRKY transcription factor response to cold stress.

Studies have shown that WRKY transcription factors are broadly involved in various biological processes. For example, *AT1G20450*, *AT1G20440*, *AT2G42540*, and *AT5G15970* are all cold-regulated genes [39]. *AT2G36270* participates in ABA signal transduction during seed maturation and germination and positively responds to drought stress [43,44]. *AT1G67320*, *AT5G04550*, and *AT2G44140* exhibit enzymatic activity [45]. *AT2G39350* was identified as an ABC transporter and is involved in substrate transport processes [46]. *AtCOR47*, *AtABI5*, *AtRAB18*, *AtCOR15A* and *AtERD10* are reported cold stress response genes [39]. The ICE-CBF-COR cascade is a critical regulatory pathway in plant cold tolerance. This cascade pathway contains three principal components, which synergistically enhance plant cold tolerance [47]. Phylogenetic analysis indicates that the *CBF* gene structure is highly conserved across both monocotyledons and dicotyledons [48]. Research indicates that *CBF* genes regulate cold stress responses by binding to the promoter region of the *COR15A* gene in Arabidopsis [49]. Overexpression of *CBF/DREB* enhances cold tolerance in Arabidopsis [50]. Numerous transcription factors can mediate the regulation of the ICE-CBF-COR cascade. For instance, overexpression of *MYB15* leads to reduced expression of CBF genes, thereby diminishing Arabidopsis cold tolerance [51]. In wheat, *TaMYC2* interacts with *TaICE41* to activate the downstream CBF-COR pathway, thereby enhancing the plant’s cold tolerance [47]. This study showed that overexpression of *TrWRKY41* significantly upregulated the expression levels of *AtCOR47*, *AtCOR15A* and *AtERD10*, thereby enhancing the cold tolerance of Arabidopsis (Figure 4). It is hypothesized that *TrWRKY41* may improve Arabidopsis cold tolerance by activating the ICE-CBF-COR cascade. Based on the above, we have constructed a schematic diagram illustrating the mechanism through which WRKY TFs regulates downstream genes in response to cold stress (Figure 5).

WRKY transcription factors serve as essential components in plant signal transduction pathways. The introduction of *TrWRKY41* into Arabidopsis can significantly improve cold tolerance. The results of target gene prediction further improved the mechanism of *TrWRKY41* in the model organism *Arabidopsis thaliana*. This study provides new candidate genes for research on the cold tolerance of white clover, and enrichment of germplasm resources. Although this study investigated the function of *TrWRKY41* after its heterologous expression in Arabidopsis plants, due to differences in the genetic background and physiological characteristics between Arabidopsis and white clover, the mechanism of action of TrWRKY41 in its native organism remains to be explored. Furthermore, this study was limited to laboratory conditions, which differ from the complex field environment. Future research will further deepen the functional investigation of the *TrWRKY41* gene in white clover.

## 4. Materials and Methods

### 4.1. Plant Material and Growing Conditions

Uniformly sized and full grains of white clover (cultivar Haifa, purchased from Barenbrug China Ltd. Com. Beijing, China) were selected as the experimental material. Seeds were vernalized at 4 °C for 2–3 days to enhance germination rates. Before sowing, the seeds were surface-sterilized with 75% (*v*/*v*) alcohol and 10% (*v*/*v*) sodium hypochlorite. Germinated seeds were sown in a substrate of perlite and sand at a volume ratio of 3:1 [27]. All seedlings were then placed in an environmental growth chamber set to a light intensity of 100 µmol/(m^2^·s) and a 16 h (24 °C): 8 h (18 °C) cycle. They were irrigated with Hoagland nutrient solution every 2 days until the second leaf was fully expanded.

To validate the effect of WRKY transcription factors on cold tolerance in white clove, four-week-old seedlings were randomly divided into four groups and treated at 4 °C for 0 min, 30 min, 1 h, and 3 h [52]. Leaves were subsequently collected. Each sample comprised five seedlings, and all samples were frozen in liquid nitrogen and stored at −80 °C.

*Arabidopsis thaliana* Columbia was selected as wild-type growth for genetic transformation. The similar growth protocol was adopted in Arabidopsis growth, such as light intensity and temperature condition. Seedlings were irrigated with distilled water every 3–4 days. Four weeks after germination, switch to watering with Hoagland nutrient solution every two weeks.

### 4.2. Isolation and Cloning of the TrWRKY41 Gene

Total RNA of white clover was detected from the leaf samples using an RNAprep Pure PlantKit (Tiangen Biotech Co., Ltd., Beijing, China). The RNA was then reverse transcribed into cDNA by PrimeScript RTkit (Dongbao, Shanghai, China), and the obtained cDNA templates were divided and stored at −20 °C in the fridge. Based on the nucleotide sequence of *TrWRKY41*, a pair of primers *TrWRKY41*-F: AAACTGCGTCAACAATTTTT and *TrWRKY41*-R: CTTGTCCACAGTGCAATACAT was designed using Primer3 (version 4.1.0) software. These primers were used to amplify the full-length *TrWRKY41* cDNA by PCR. The purified PCR product was ligated into the pMD™18-T Vector using the Cloning Kit and sequenced. NCBI BLAST (https://blast.ncbi.nlm.nih.gov/Blast.cgi) (accessed on 25 August 2024) software were utilized to compare the homologous amino acid sequences of TrWRKY41 proteins. To exclude the influence of Arabidopsis homologous genes, the MEGA11 (version 11.0) software was used to construct a phylogenetic tree of TrWRKY41 and the Arabidopsis WRKY gene family. The phylogenetic tree was constructed using the neighbor-joining (NJ) method with the bootstrap replicates set to 1000 [53].

### 4.3. Construction and Transformation of Plant Expression Vectors

Both the pMD18T-*TrWRKY41* plasmid and the pCAMBIA1300 plasmid were double-digested with *Pst*I/*Bam*HI. The digested fragments were then separately purified. The digested fragments were ligated using T4 DNA ligase at 16 °C for 4–5 h. The ligation product was then transformed into *E. coli* DH5α competent cells by the heat-shock method. After overnight incubation in LB medium containing kanamycin antibiotic, single colonies that were successfully identified were picked and incubated on a shaker at 37 °C at 200 rpm for 12 h. The plasmid was extracted and sequenced, and the correct plasmid was named pCAMBIA1300-*TrWRKY41*. The recombinant plasmid was introduced into Agrobacterium tumefaciens GV3101 by freeze–thaw method.

Four-week-old Arabidopsis plants were selected, and the already-flowering inflorescences and pods were removed with shears. The unflowering but whitish inflorescences were sequentially immersed in the resuspension solution for about 30 s [54]. Following watering, plants were incubated in darkness for 24 h before being transferred to standard light growth conditions. T0 generation seeds were subsequently harvested. Transformant seeds were germinated in MS (0.5×) medium (containing 50 mg/L kanamycin) to identify tolerance plants. After 14 days, the healthy seedlings were transferred to the above greenhouse soil conditions for further cultivation. The DNA of Arabidopsis leaves was used as template for PCR validation. The samples with amplified products of the expected size were sent to BGI Genetics for sequencing. The above methods were used until the T3 generation positive transgenic plants were selected for the experiment. T3 generation positive plants and wild-type Arabidopsis were sown in nutrient soil at a ratio of 1:1 and watered regularly with Hoagland nutrient solution (Solution A: 0.945 g/L, Solution B: 1.240 g/L).

### 4.4. Measurement of Relevant Physiological Indicators

Wild-type and homozygous T3 lines Arabidopsis seeds were sterilized and cleaned. Seeded in solid MS (0.5×) medium, vernalized for 3 days at 4 °C, then grown in a light chamber for four weeks, see previous described [55]. There were two chambers in Arabidopsis analysis, one chamber was set as control condition, with 24/18 °C across experiments, and another chamber was cold stress condition, with four weeks normal condition, and the temperature was set as 4 °C for simulating cold stress. In each chamber, there were five groups of Arabidopsis in total, three independent transgenic lines and two control groups (all of them were WT Arabidopsis lines). After four-weeks, the control condition was set with normal temperature, while cold stress chamber was set with 4 °C, 24 h later, considering cost of experiments, four groups of Arabidopsis from each chamber, one WT group and three transgenic lines, were harvested for RNA detection and physiological analysis. All samples were then rapidly frozen in liquid nitrogen and stored in a −80 °C freezer. qRT-PCR analysis was used to measure the expression of the *TrWRKY41* gene in homozygous lines. The reaction conditions and system for qRT-PCR followed the operating procedures of SYBR PreMix Ex Taq™ II (Toyobo, Shanghai, China), and three biological replicates per group GADPH is used as an internal reference gene. Use the T-test for intergroup comparisons (** *p* ≤ 0.01). Expression data were calculated using the 2^−ΔΔCT^ method [56].

In addition, the above-mentioned Arabidopsis samples were also used to the determination of chlorophyll (CHL), proline (Pro), malondialdehyde (MDA), catalase (CAT), peroxidase (POD), superoxide dismutase (SOD) content. The Chlorophyll content was determined using the acetone extraction method [57]. The MDA content, an indicator of lipid peroxidation, was measured using the thiobarbituric acid (TBA) method. Briefly, fresh leaf sample was ground in liquid nitrogen and homogenized in 0.9 mL of extraction buffer containing 50 mM phosphate buffer (pH 7.4) and 1% polyvinylpyrrolidone (PVP). The homogenate was centrifuged at 3500 rpm for 10 min at 4 °C. The supernatant was collected, and its absorbance was measured at 532 nm using a UV spectrophotometer (Thermo, Halios Beta, Waltham, MA, USA). The MDA content was calculated according to the standard protocol [58]. For the extraction of SOD and POD, leaf sample was ground in liquid nitrogen using a pre-chilled mortar and pestle, followed by homogenization in 3.0 mL of extraction buffer consisting of 50 mM phosphate buffer (pH 7.8) and 1% PVP. The homogenate was centrifuged at 10,000 rpm for 20 min. The resulting supernatant was used for enzyme activity assays. SOD activity was determined according to the method of Li et al. (2013), and one unit SOD activity (U) was defined as the quantity of SOD required to produce 50% inhibition of the reduction of nitrite in 1 mL reaction solution by measuring the change of absorbance at 550 nm [58]. POD activity was assayed in a reaction mixture containing 50 mM phosphate buffer (pH 7.0), 0.2% guaiacol, 0.3% H_2_O_2_, and enzyme extract. The activity was measured by monitoring the increase in absorbance at 470 nm due to guaiacol oxidation. One unit of POD activity was defined as an increase in absorbance of 0.01 per minute [59]. CAT activity was determined by tracking the decrease in absorbance at 405 nm resulting from H_2_O_2_ decomposition via the molybate method. One unit of CAT activity was defined as the amount of enzyme that decomposes 1 μmol of H_2_O_2_ per second [58]. All enzyme activities were measured using a microplate reader (Thermo, Halios Beta, Waltham, MA, USA). Each experiment was performed with three independent replicates. To compare differences within and between groups, statistical analysis was performed on the data. Analysis of variance (ANOVA) was performed using R (version 4..4.2) software, followed by Duncan’s test (*p* < 0.05) for assessment. Different letters indicate significant differences.

### 4.5. Identification of TrWRKY41 Transcription Factor (W-Box) and Target Gene Prediction

To further reveal the regulatory mechanisms of WRKY transcription factors on downstream target genes in Arabidopsis, we performed a whole-gene scan and comparison of Arabidopsis promoter regions. First, the Arabidopsis upstream 2000 bp sequence was downloaded from the TAIR database (https://www.arabidopsis.org/). (accessed on 11 March 2025) These sequences were defined as promoter regions. W-box elements are short and highly conserved. Therefore, the regular expression matching algorithm is used to identify proteins containing the core sequence TTGAC [C/T]. The 20 most frequently occurring motifs were selected as representative W-box elements. To enhance prediction accuracy and match proteins with complete W-box elements, we extended the upstream and downstream regions of the element by 4 bp (with downstream padding as needed) based on miRNA length (20–24 nt), resulting in a 17-bp extended sequence [60]. Subsequently, the extracted Arabidopsis promoter regions were subjected to BLAST alignment, using an E-value threshold of 2 to find downstream target genes [61]. Initial alignments permitted a sequence length variation of 2–6 nt and could tolerate a maximum of two internal mismatches [62]. Based on the comparison results, 30 sequences containing W-box elements were randomly selected, and their potential interactions with TrWRKY41 were predicted using AlphaFold2 (version 0.2.0) software [63]. Results were visualized using Chimera X (version 1.9) software [64]. In addition, to comprehensively analyze *TrWRKY41* function, downstream target genes were functionally classified based on annotation data from the TAIR database.

### 4.6. Expression Analysis of WRKY-Regulated Downstream Target Genes in Arabidopsis

Total RNA was extracted from WT and transgenic lines using the RNA pure Plant Kit (Tiangen, Beijing, China), as Section 4.4 described. Subsequently, cDNA was synthesized using the PrimeScript RT kit (Toyobo, Shanghai, China) and used as a template for quantitative reverse transcription PCR (qRT-PCR). All operations were performed according to the manufacturer’s instructions. Six candidate genes, namely *AtCOR47*, *AtCOR6.6*, *AtABI5*, *AtRAB18*, *AtCOR15A*, *AtERD10*, were selected for further validation, and six pairs of primers were designed based on their nucleic acid sequences (Table A1). Each biological sample performed three technical replicates. The results were normalized. Subsequently, the T-test was used to compare the groups, with asterisks indicating significant differences between groups (** *p* ≤ 0.01). Three replicates were set for each sample. Expression data were calculated using the 2^−ΔΔCT^ method [56].

## 5. Conclusions

In this study, we successfully cloned the *TrWRKY41* gene of white clover. The biological function and mechanism of this gene in response to low temperature stress in Arabidopsis were revealed. TrWRKY41 protein is an unstable acidic protein, mainly distributed in the nucleus, and is a hydrophilic protein. Compared to WT plants, *TrWRKY41*-overexpressing plants exhibited significantly elevated chlorophyll and proline content, markedly enhanced antioxidant enzyme activity, and substantially reduced malondialdehyde levels. This indicates that TrWRKY41 enhances Arabidopsis cold tolerance by mitigating cell membrane damage, regulating osmotic pressure, and boosting antioxidant capacity. AlphaFold2 prediction results indicate that the *TrWRKY41* gene can interact with the W-box element of target genes, thereby performing specific biological functions. Furthermore, qRT-PCR results indicate that the expression levels of cold-response genes (such as *AtCOR15A* and *AtERD10*) were increased in the overexpressing plants. It is hypothesized that the *TrWRKY41* gene enhances Arabidopsis cold tolerance by participating in the ICE-CBF-COR cascade. This study fully analyzed the physicochemical properties of the *TrWRKY41*, aiming to understand its cold tolerance mechanism from structural and functional perspectives. Furthermore, this analysis provides insights enabling deeper exploration of WRKY TFs functions.

## Figures and Tables

**Table 1:** Prediction of binding sites between TrWRKY41 and target genes.

Downstream Target Genes	Sequences	Reverse Complemented Sequences	ipTM
*AT5G04550*	ACGAACTTGACTCGCTC	GAGCGAGTCAAGTTCGT	0.86
*AT1G35435*	TATGAAGCTTGACTAAC	GTTAGTCAAGCTTCATA	0.85
*AT2G04630*	CTGAACTTGACTCGAAA	TTTCGAGTCAAGTTCAG	0.85
*AT1G09570*	TCATATTTGTTTTGTTC	GAACAAAACAAATATGA	0.84
*AT1G20450*	TCCAATCTGTTTCTTCT	AGAAGAAACAGATTGGA	0.84
*AT1G67320*	TAGTGATAGTCAACTTG	CAAGTTGACTATCACTA	0.83
*AT1G20800*	GAAGACGAGTCAAGTAG	CTACTTGACTCGTCTTC	0.83
*AT1G20440*	GAGTGACCACCCCAATG	CATTGGGGTGGTCACTC	0.86
*AT1G21240*	CACAGCTTGACTCATAT	ATATGAGTCAAGCTGTG	0.83
*AT1G20810*	TCTACTTGACTCGTCTT	AAGACGAGTCAAGTAGA	0.82
*AT1G44170*	AATTATTTGTTTTGTTA	TAACAAAACAAATAATT	0.82
*AT2G40910*	CCTGCAGTTACGATGAT	ATCATCGTAACTGCAGG	0.82
*AT2G39350*	AGATAACTTGACTCGAA	TTCGAGTCAAGTTATCT	0.82
*AT2G47790*	AAAAGTTGACTATCATT	AATGATAGTCAACTTTT	0.82
*AT5G57530*	ATGATTGACTGAGATTT	AAATCTCAGTCAATCAT	0.82
*AT5G47910*	AAAGGATTTTGACCAGA	TCTGGTCAAAATCCTTT	0.82
*AT1G32750*	GATATTTGTTTTGTTTG	CAAACAAAACAAATATC	0.81
*AT1G63500*	ATAACTAGTTATATGAA	TTCATATAACTAGTTAT	0.81
*AT2G42540*	TCTATACCACTGTAAAA	TTTTACAGTGGTATAGA	0.81
*AT1G47480*	ATCAAAGCTTGACTCTC	GAGAGTCAAGCTTTGAT	0.81
*AT5G17970*	TAAAAGCTTGACTCAGC	GCTGAGTCAAGCTTTTA	0.81
*AT5G44580*	AACAGCTTGACTCAGAC	GTCTGAGTCAAGCTGTT	0.80
*AT2G44140*	CGTAACTTGACTCGATA	TATCGAGTCAAGTTACG	0.80
*AT5G35580*	CTAGAAGCTTGACTCGC	GCGAGTCAAGCTTCTAG	0.80
*AT5G13320*	AAAACATTGACCCAGAC	GTCTGGGTCAATGTTTT	0.80
*AT5G57540*	TATGATTGACTGAGATT	AATCTCAGTCAATCATA	0.80
*AT2G36270*	TCACAAAACAAATCATG	CATGATTTGTTTTGTGA	0.80
*AT5G15970*	TCAACAAATATACAACT	AGTTGTATATTTGTTGA	0.80
*AT1G43890*	AGGTTGGATTTTGATCG	CGATCAAAATCCAACCT	0.80

**Figure A3:**
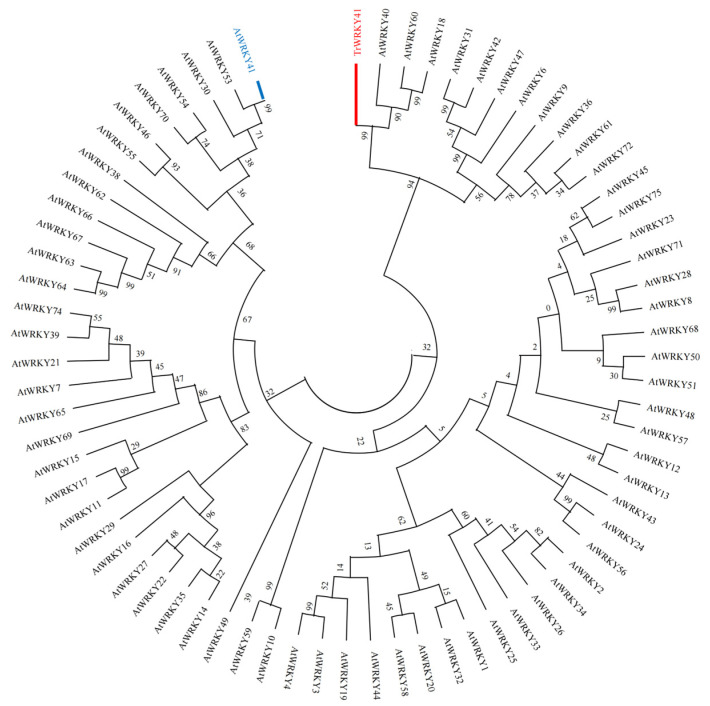
Phylogenetic analysis of Arabidopsis WRKY gene family members and TrWRKY41. The phylogenetic tree was constructed using the neighbour-joining (NJ) method and subjected to 1000 bootstrap iterations. Among them, the AtWRKY41 and TrWRKY41 proteins are represented by blue and red, respectively.

## Data Availability

The datasets presented in this study can be found in Appendix A and Appendix B.

## Appendix A

### Appendix A.1

**Table A1:** Primers for *TrWRKY41* and target genes.

Primer Name	Primer Sequence (5′-3′)
*TrWRKY41*-F	AAACTTGCGTCAACAATTTTT
*TrWRKY41*-R	CTTGTCCACAGTGCAATACAT
q*TrWRKY41*-F	GCTATGCTATTGCTGAGAGC
q*TrWRKY41*-R	CCTAGGAGAGGGGTTATCTC
q*AtCOR47*-F	GAAACCTCAAGAGACAACGA
q*AtCOR47*-R	AGAAGAGCTGTTGGATCGG
q*AtCOR6.6*-F	AGAGACCAACAAGAATGCC
q*AtCOR6.6*-R	TGTCCTTCACGAAGTTAACAC
q*AtABI5*-F q*AtABI5*-R	TCCAGTGGAGAAAGTAGTGG AGGTGTCTTTACCTGTTGCT
q*AtRAB18*-F	GTCAGGACAACCCGAATTT
q*AtRAB18*-R	ATACGAACTTGTTAGCGTCC
q*AtCOR15A*-F	GAACAAGCCTAGTGTCATCG
q*AtCOR15A*-R	ATCATCCTCTGCTGTCTTGT
q*AtERD10*-F	ACATTCGGTAGAGGATCACA
q*AtERD10*-R	TTCCTCTCCAGTGGTCTTG

### Appendix A.2

**Table A2:** Top 20 most frequent W-box extended motifs (17 bp).

Motif Name	Sequence (5′-3′)
W-box 1	AAAGGATTTTGACCAGA
W-box 2	ATTTATTTGTTTTGTTT
W-box 3	CAAAGTTGACTATCATT
W-box 4	AGTGGATTTTGACCATG
W-box 5	ATTGATTGACTGAGAAT
W-box 6	AGTAGTTGACTATCACA
W-box 7	CTCCATTGACCCAGAGA
W-box 8	GTTACTTGACTCGAAAC
W-box 9	GATGAAGCTTGACTCAA
W-box 10	GTTAGGATTTTGACCAC
W-box 11	TCTTGTTCGATTGACCG
W-box 12	TAACTAGTTATATAATG
W-box 13	ACGTTTTGACCACTGTT
W-box 14	CTATTCGATTGACCAAA
W-box 15	TTGATACATTGACCCAT
W-box 16	GATAACTTGACTCGTCT
W-box 17	TTTTACATTGACCCCAG
W-box 18	TTAATTTTGACCACTCA
W-box 19	GGAAATTTTGACCACTG
W-box 20	TACAATTGACAATCTTT
